# Contralateral Adaptations After Unilateral Power Training in Older Adults: The Effect of Intensity of Load

**DOI:** 10.1111/sms.70165

**Published:** 2025-11-12

**Authors:** Carlos Rodriguez‐Lopez, Julian Alcazar, Coral Sanchez‐Martin, Ivan Baltasar‐Fernandez, Ignacio Ara, Robert Csapo, Luis M. Alegre

**Affiliations:** ^1^ Department of Geriatrics Hospital General Universitario Gregorio Marañón, Health Research Institute Gregorio Marañón Madrid Spain; ^2^ Centro de Investigación Biomédica en Red Fragilidad y Envejecimiento Saludable (CIBERFES) Instituto de Salud Carlos III Madrid Spain; ^3^ GENUD Toledo Research Group, Faculty of Sport Sciences University of Castilla‐La Mancha Toledo Spain; ^4^ Grupo Mixto de Fragilidad y Envejecimiento Exitoso UCLM‐SESCAM Universidad de Castilla‐La Mancha‐Servicio de Salud de Castilla‐La Mancha, IDISCAM Toledo Spain; ^5^ Faculty of Health Sciences University of Castilla‐La Mancha Talavera de la Reina Spain; ^6^ Department of Sport and Human Movement Science, Centre for Sport Science and University Sports University of Vienna Vienna Austria

**Keywords:** cross‐education, cross‐transfer, immobilization, rehabilitation, speed, strength, ultrasound

## Abstract

This study aimed to investigate the effect of training intensity on contralateral adaptations following unilateral power‐oriented resistance training (PT) in older adults. This secondary analysis of a within‐person randomized controlled trial included 45 older adults (≥ 65 years; 25 women). After an 8‐week control period (*n* = 45), part of the participants completed 12 weeks of a volume load‐matched PT on one lower limb using either light loads (40% 1‐RM, *n* = 9) or heavy loads (80% 1‐RM, *n* = 10), while the contralateral limb did not exercise. Unilateral performance was assessed before and after each period through isometric tests (maximal isometric force, rate of force development, and muscle excitation) and dynamic tests (1‐RM, maximal muscle power and maximal unloaded velocity determined through a force‐velocity relationship test). Additionally, mid‐thigh muscle mass and whole‐body physical function were assessed. Linear mixed models were conducted to compare the effect (Cohen's *d*) of training intensity on the efficacy and magnitude of the contralateral adaptations and their impact on physical function. No contralateral adaptations were observed in any of the isometric tests. In contrast, untrained limbs showed comparable improvements in 1‐RM (*d* = 0.35–0.42; *p* ≤ 0.001) and maximal muscle power (*d* = 0.49–0.54; *p* ≤ 0.001) regardless of PT intensity. Only heavy‐load PT improved maximal unloaded velocity in the contralateral untrained limb (*d* = 0.68; *p* ≤ 0.001), while mid‐thigh muscle mass (*d* = 0.24; *p* ≤ 0.013) and whole‐body physical function assessed through the timed up‐and‐go test improved only after light‐load PT (*d* = −0.74; *p* < 0.001). In conclusion, unilateral PT induced significant contralateral gains in dynamic muscle strength and power, regardless of training intensity, with heavier loads enhancing velocity‐specific adaptations. Notably, light‐load PT induced mild hypertrophy in the contralateral untrained limb and whole‐body physical function, which might be particularly relevant for enhancing the intrinsic capacity of older adults.

**Trial Registration:** ClinicalTrials.gov ID: NCT03724461

## 1 Introduction

The aging process is related to a progressive loss of muscle mass and function that contributes to the reduction of intrinsic capacity (i.e., a composite of all physical and mental capacities) of older people [1, 2]. In addition, a dramatic episode of hospitalization or immobilization might accelerate this deleterious process, exposing weaker individuals to further limitations in mobility and activities of daily living [3]. Suetta et al. [4] illustrated how a 2‐week leg immobilization produced meaningful declines in voluntary activation (−10%), maximal isometric force (−20%) and muscle volume (−5%) in older adults. Moreover, the restoration of baseline values required twice as much time for retraining; except for muscle volume, for which 4 weeks of retraining was not enough. In this sense, unilateral training of the able limb would be useful for maintaining muscle strength and size after immobilization by means of the cross‐education phenomenon (CE) [5, 6]. This phenomenon refers to the increased motor output (i.e., force generation, skill) of the opposite untrained limb following a period of unilateral exercise training [7]. In this case, unilateral resistance training is the most extended training type to induce CE in older adults [6].

Among resistance training guidelines for older adults [8], the inclusion of power‐oriented exercises (i.e., the concentric phase is performed as fast as possible) is strongly recommended, since muscle power declines faster and is more closely related to physical function than muscle strength [8, 9, 10]. In contrast to strength‐related CE, whether muscle power could be developed in a non‐exercised limb after unilateral power training (PT) conducted in the contralateral limb has been poorly investigated in older adults [11, 12]. Hester et al. presented a couple of studies supporting that age does not mitigate the CE of rapid contractile properties (i.e., maximal contraction velocity and rate of force development) yielded by unilateral resistance training [11, 12]. However, the influence of different resistance training variables on CE of rapid contractile properties (e.g., maximum contraction velocity or maximal muscle power) has not been explored in older adults. In terms of training intensity (i.e., % one‐repetition maximum, %1‐RM), light‐moderate to heavy intensities (i.e., 40 vs. 80% 1‐RM, respectively) appear to be equally effective for improving muscle power and physical function in older adults [13], even though specific adaptations over force‐ and velocity‐dependent components of power have been reported [14]. The body of evidence about the effect of intensity on CE magnitude, mostly conducted on young adults, points to superior effects when using higher vs. lower loads [15]. Nevertheless, the effect of intensity on CE in older populations after PT is not well understood. Ascertaining the most effective training intensity that maximizes CE after unilateral PT would help in the design of exercise‐based interventions such as those aimed at minimizing the impact of limb immobilization in older adults. Moreover, given the importance of physical performance in activities of daily living for the quality of life and independence of older adults, it may be reasonable to examine whether the potential benefits of CE over muscle function transfer to functional performance in such activities. Accordingly, this study aimed to explore the potential CE induced after 12 weeks of light versus heavy load unilateral PT in older adults in terms of muscle strength, power, muscle mass and functional performance. In light of existing literature, we hypothesized that unilateral PT with heavy loads (80% 1‐RM) might elicit greater CE effects on muscle strength, power and functional performance in older adults compared to light loads (40% 1‐RM).

## 2 Methods

### 2.1 Study Design

**FIGURE 1:**
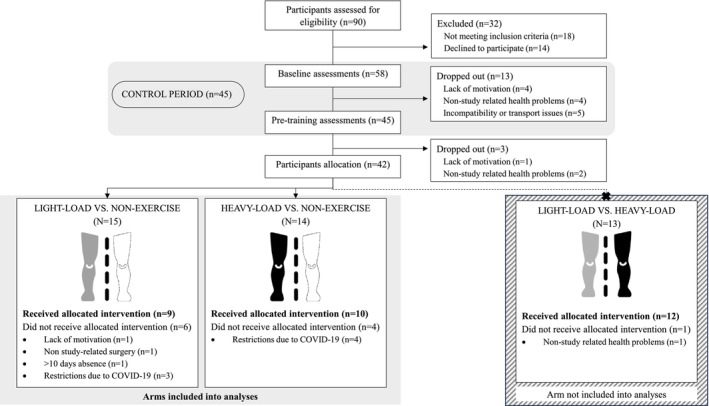
Participant selection, allocation and dropouts.

The present study is a secondary analysis of a randomized controlled trial previously described and registered in ClinicalTrials.gov (October 30th, 2018) [16]. The study was performed as a within‐person randomized controlled trial with repeated measures and included an 8‐week control period (CTR) followed by 12 weeks of PT targeting the lower limbs. For the training period, participants were randomly allocated to one of the following study arms: (i) one leg performed light‐load PT (LL‐PT) and the contralateral leg did not perform any exercise (LL‐NE); (ii) one leg performed heavy‐load PT (HL‐PT) and the contralateral leg did not perform any exercise (HL‐NE); and (iii) one leg performed LL‐PT and the other leg performed HL‐PT (Figure 1). For this secondary analysis, only the study arms (i) and (ii) were included in the analysis, as they were the only groups including a non‐exercise leg (LL‐NE and HL‐NE, respectively). The exercised leg within each study arm was randomized by a coin toss. Detailed information about the study design was provided elsewhere [16].

### 2.2 Participants

Volunteers aged 65 or older were recruited through local advertisements or by contacting participants from non‐exercise interventions previously conducted in our laboratory. On those who accepted to participate, a physical examination was conducted by a geriatrician who screened the following exclusion criteria: frailty or low physical function (i.e., SPPB score ≤ 7 points), history of regular resistance exercise training in the last 3 years or knee arthroplasty. Participants' selection and allocation, as well as the reasons for dropouts are shown in Figure 1. A priori sample size calculation and further details about the study design have been published elsewhere [16]. All participants signed a written informed consent about the risks and benefits of participation. This study was conducted in accordance with the Helsinki Declaration and approved by the Clinical Research Ethics Committee of the Complejo Hospitalario de Toledo (Spain) (ref. 25012017).

### 2.3 Interventions

Both PT interventions were carried out on a horizontal leg press device (Selection MD, Technogym, Italy), and targeted the lower limbs unilaterally twice a week, with at least 48 h of rest between sessions. LL‐PT consisted of 6 sets of 12 repetitions with a load equivalent to 40% 1‐RM, whereas HL‐PT consisted of 6 sets of 6 repetitions with a load equivalent to 80% 1‐RM. Hence, the volume × load (i.e., number of repetitions × external load relative to 1‐RM) was matched between programs. To isolate the potential effect of intensity, the volitional effort exerted by the participants in all study arms was matched according to the following rules. For both intensities, the participants were asked to execute the concentric phase of each repetition as fast as possible (i.e., ballistically). For the eccentric phase, the participants were required to lower the weights in 3 and 2 s during LL‐PT and HL‐PT, respectively. Note that according to the force‐velocity relationship during eccentric contractions, lengthening velocity increases as a function of force [17]. Thus, achieving comparable volitional efforts under heavy and light loading conditions requires faster eccentric velocities (i.e., shorter eccentric duration) during the former. The timing for the eccentric phase was controlled verbally with the corresponding cue (“3, 2, 1, go!” for LL‐PT and “2, 1, go!” for HL‐PT, with the “go!” cue denoting the start of the concentric phase of the following repetition), and the participants were previously familiarized with this movement pattern. A 2‐min passive recovery was granted between sets. The training loads were revised every 4 weeks by conducting a 1‐RM test to ensure progressive overload. The principal mechanical characteristics of LL‐PT and HL‐PT have been published elsewhere [18]. Participants must have attended at least 80% of the scheduled training sessions to be included in the analysis. All the training sessions were supervised individually by sport scientists who were part of the research team.

### 2.4 Procedures

The participants were evaluated on three different occasions: week 0 (W0), week 8 (W8) and week 20 (W20). The interval between W0 and W8 corresponded to the 8‐week CTR period whereas the interval between W8 and W20 corresponded to the 12‐week PT period. The standardized protocol followed to evaluate the main outcomes of this study has been previously published [16]. Briefly, the neuromuscular performance was assessed unilaterally through an isometric test for the knee extensors and a dynamic progressive loading test for the lower limbs. The former was conducted on a custom‐built rigid chair (Telju Fitness, Alcabon, Spain) instrumented with a strain gauge (Linear Force‐SmartLead, Noraxon, USA; 1500 Hz). The test consisted of several unilateral maximal voluntary isometric contractions (MVIC) of the knee extensors and flexors at a knee flexion of 90° (0° = full extension). Accordingly, the maximal isometric force (MIF) and rate of force development (RFD) in the first 50, 100 and 200 ms (RFD_50_, RFD_100_ and RFD_200_, respectively) after the onset of contraction were determined. Muscle excitation of the quadriceps muscle (including rectus femoris, vastus medialis and vastus lateralis muscles) and the long head of the biceps femoris muscle was registered during the MVIC using surface electromyography (sEMG) captured at 1500 Hz, amplified and filtered with a band‐pass filter between 10 and 500 Hz (common mode rejection ratio > 100 dB, input impedance > 100 MΩ, and gain = 500) (DTS EMG sensors and Desktop DTS, Noraxon, USA). sEMG‐derived signals were integrated and normalized to the maximal value registered at MIF (% iEMG) during the best corresponding MVIC. Then, the quadriceps % iEMG acquired during the first 50, 100 and 200 ms of contraction (QiEMG_50_, QiEMG_100_ and QiEMG_200_, respectively) was determined together with the corresponding biceps femoris co‐activation [16]. An automated threshold method was used to detect the onsets of muscle excitation and force production as the instant when each signal exceeded a level corresponding to the mean plus 3 SD of their respective baseline noise levels. Thereafter, the unilateral progressive loading test was conducted on a horizontal leg press device equipped with a linear position transducer (Linear encoder, Chronojump Bosco System, Spain; 1019 Hz) and a force plate (Type 9286BA, Kistler, Switzerland; 1000 Hz). During this test, the participants were asked to extend their lower limb as fast as possible against progressive loads until achieving the 1‐RM. On average, 5.0 ± 0.8 loads were used, requiring a minimum of 3 loads, in increments ranging from 5 to 20 kg, starting at a load corresponding to their ~40% body mass. The force‐velocity relationship (*F*–*V*) was determined by fitting the best attempts of each load using a linear model and the estimated maximal isometric force (*F*_0_—force intercept) and maximal unloaded velocity (*V*_0_—the velocity intercept) were calculated, as well as the slope of the *F*–*V* relationship (*S*_FV_) and the maximum power output (*P*_max_) [19]. Additionally, the participants' functional performance was evaluated through the 30‐m maximal gait speed test (MGS), the 3‐m timed up and go test (TUG) and the 5‐repetition sit‐to‐stand test (5‐STS). The mean concentric power during the 5‐STS was estimated according to a previously validated equation [20, 21] and normalized to participants' body mass. Finally, an ultrasound examination (MyLab 25, Esaote Biomedica, Italy; 50 mm linear array probe, 10–15 MHz) was performed in the mid‐thigh region (50% of the femur length) for each of the participants' legs. Transversal images were obtained using the extended field of view feature of the scanner to determine the sum of the cross‐sectional area of the rectus femoris and vastus lateralis muscles (*Q*_CSA_). Images were analyzed with FIJI, an open‐source software for biomedical image analysis [22].

### 2.5 Statistical Analysis

Data are presented as means ± standard deviation unless otherwise stated. The Shapiro–Wilk test and normal probability plots were used to evaluate the normal distribution of the data, which were log‐transformed in case of non‐normal distribution. The absolute and relative changes observed in each of the participants' legs after the CTR (i.e., W8 minus W0) and PT (i.e., W20 minus W8) periods were used in the statistical analysis. We therefore assessed the changes that occurred after the 8‐week CTR and following the 12‐week LL‐PT or HL‐PT, focusing on the exercised legs as well as examining any CE in the contralateral non‐exercised legs (i.e., LL‐NE and HL‐NE, respectively). To control for partially correlated data (e.g., two legs of the same participant contributing to two different treatment groups) and handle missing data (e.g., legs that only completed the CTR period), the analyses were performed using a linear mixed‐effect model [23]. The applied treatment (i.e., CTR, LL‐PT, LL‐NE, HL‐PT, and HL‐NE) was included as a fixed factor, participants as a random factor, and baseline values and the duration of the corresponding period (i.e., CTR or PT) as covariates. For the functional performance variables, the treatments included as fixed factors were limited to CTR and the PT applied (i.e., LL‐PT and HL‐PT), since physical performance tests involved using both legs. The maximum likelihood estimation and the best‐fitting covariance structure were considered for the model according to the chi‐square likelihood ratio test. Given that most models included only the random intercept with one level, the variance components structure was most frequently preferred as the best‐fitting covariance structure [24]. To explore differences between treatments, pairwise Bonferroni‐adjusted comparisons were conducted. Additionally, the magnitude of treatment effects was determined by the Hedges' *g* effect sizes and classified as trivial (< 0.20), small (0.20–0.49), moderate (0.50–0.79), and large (≥ 0.80). Of note, effect sizes were calculated as the between‐group differences (found by the linear mixed‐effect models) normalized to the SD values reported by the whole group of participants, and further adjusted by the sample size according to Hedge's *g*. All statistical analyses were performed using SPSS Statistics 24 (IBM Corp, Armonk, NY), and the level of significance was set at *α* = 0.05.

## 3 Results

The baseline characteristics of the study participants are summarized in Table 1. By the end of this study, the total number of participants (women) was 45 (25) for CTR, 9 (5) for study arm I (LL‐PT vs. non‐exercise) and 10 (5), for study arm II (HL‐PT vs. non‐exercise). The mean age of the participants was not significantly different between groups (*p* = 0.648). Similarly, there were no significant differences in baseline body composition or functional performance between groups (*p* > 0.05 for all comparisons). No differences were found in the load progression made between HL‐PT and LL‐PT after the first 4 weeks of training (relative load increase: +18.8% ± 16.1% vs. +15.3% ± 12.4%, respectively; *p* = 0.608) nor after 8 weeks of training (+6.7% ± 12.6% vs. +8.2% ± 7.5%, respectively; *p* = 0.758).

**TABLE 1:** Baseline characteristics of study participants.

	CTR*		LL‐NE		HL‐NE		*p*
	(*n* = 45; 25 women)		(*n* = 9; 5 women)		(*n* = 10; 5 women)		
	Mean (SD)	Range	Mean (SD)	Range	Mean (SD)	Range	
Age (years)	70.6 (4.2)	(64.0–83.0)	70.7 (5.5)	(65.0–83.0)	70.2 (4.2)	(64.0–79.0)	0.648
Weight (kg)	77.0 (16.9)	(51.4–130)	67.3 (7.2)	(57.1–78.6)	75.7 (16.8)	(51.4–104)	0.335
Height (cm)	163 (8.0)	(149–180)	161 (7.3)	(149–173)	167 (9.5)	(153–180)	0.390
BMI (kg/m^2^)	28.9 (5.3)	(20.2–48.0)	26.1 (2.3)	(22.8–30.6)	27.1 (4.4)	(20.2–33.9)	0.141
ASM (kg/m^2^)^a^	7.4 (1.6)	(4.71–10.7)	6.9 (1.4)	(5.21–8.7)	7.1 (1.6)	(4.71–9.0)	0.767
Body fat (%)^a^	36.7 (7.8)	(14.7–48.6)	34.8 (11.1)	(14.7–46.0)	35.1 (4.4)	(28.09–42.1)	0.867
SPPB (points)	11.9 (0.3)	(11.0–12.0)	11.9 (0.3)	(11.0–12.0)	12.0 (0.0)	(12.0–12.0)	0.793
Handgrip strength (kg)	31.0 (9.2)	(18.9–49.4)	29.2 (7.4)	(23.1–44.1)	31.7 (9.6)	(18.9–42.3)	0.991
Relative STS power (W/kg)	4.3 (0.8)	(2.6–6.5)	4.2 (0.8)	(3.0–5.6)	4.3 (1.0)	(2.9–6.5)	0.799

Abbreviations: ASM, appendicular skeletal muscle index; BMI, body mass index; CTR, control period; HL‐NE, heavy‐load vs. non‐exercise; LL‐NE, light‐load vs. non‐exercise; SPPB, short physical performance battery; STS power, mean power registered during sit‐to‐stand test.

^a^ ASM and body fat were determined by Dual‐energy X‐Ray Absorptiometry. Details about these descriptive variables were previously described in the published study protocol [16].

* Note that CTR represents a control period rather than a parallel group. The subjects assigned to LL‐NE, HL‐NE, and LL‐HL constitute 19/45 participants in this column.

### 3.1 MIF and RFD on the Knee‐Extensors Isometric Test

The main effects observed on the knee‐extensors isometric test are presented in Table 2. In summary, no differences were observed for MIF, RFD or QiEMG among treatments.

**TABLE 2:** Knee‐extensor isometric neuromuscular performance.

	Treatment	PRE	Change (∆)	Time effect		Treatment effect		
		Mean (SD)	Mean (SD)	ES [95% CI]	*p*	*F*	*p*	Post hoc
MIF (N)	CTR	381 (118)	1.4 (47.6)	0.01 [−0.08, 0.11]	0.767	1.546	0.196	
	LL‐NE	368 (126)	30.6 (31.7)	0.15 [−0.08, 0.37]	0.197			
	HL‐NE	369 (169)	14.5 (47.5)	0.16 [−0.05, 0.37]	0.130			
	LL‐PT	379 (122)	34.1 (24.6)	0.21 [0.00, 0.43]	0.045			
	HL‐PT	364 (140)	8.7 (26.2)	0.11 [−0.11, 0.32]	0.326			
RFD_50_ (N s^−1^)	CTR	555 (310)	41.2 (275.0)	0.12 [−0.05, 0.29]	0.155	1.563	0.188	
	LL‐NE	686 (285)	7.3 (175.8)	0.12 [−0.44, 0.69]	0.675			
	HL‐NE	601 (440)	97.9 (190.0)	0.34 [−0.19, 0.87]	0.202			
	LL‐PT	633 (312)	213.8 (415.4)	0.74 [0.21, 1.27]	0.006			
	HL‐PT	457 (247)	−4.0 (133.6)	−0.10 [−0.64, 0.43]	0.713			
RFD_100_ (N s^−1^)	CTR	1155 (624)	−10.1 (408.0)	−0.01 [−0.13, 0.11]	0.884	0.448	0.774	
	LL‐NE	1061 (380)	56.2 (236.8)	0.07 [−0.34, 0.47]	0.756			
	HL‐NE	1100 (751)	31.4 (207.9)	0.04 [−0.34, 0.42]	0.848			
	LL‐PT	1133 (434)	136.4 (463.6)	0.22 [−0.17, 0.60]	0.266			
	HL‐PT	1036 (503)	−56.8 (357.0)	−0.12 [−0.50, 0.26]	0.546			
RFD_200_ (N s^−1^)	CTR	1131 (454)	−24.7 (271.1)	−0.05 [−0.16, 0.07]	0.428	0.238	0.916	
	LL‐NE	1063 (315)	41.1 (200.7)	0.07 [−0.31, 0.46]	0.721			
	HL‐NE	1042 (611)	−1.4 (284.7)	−0.03 [−0.39, 0.34]	0.885			
	LL‐PT	1121 (349)	29.8 (257.4)	0.07 [−0.29, 0.43]	0.720			
	HL‐PT	1034 (367)	−46.9 (246.3)	−0.13 [−0.50, 0.23]	0.483			
QiEMG_50_ (% MVIC s)	CTR	4.94 (1.6)	0.10 (1.5)	0.04 [−0.14, 0.23]	0.646	1.849	0.124	
	LL‐NE	6.03 (2.1)	−1.28 (2.3)	−0.53 [−1.14, 0.09]	0.088			
	HL‐NE	4.84 (1.9)	0.39 (1.6)	0.20 [−0.37, 0.77]	0.497			
	LL‐PT	5.03 (1.3)	0.58 (1.9)	0.36 [−0.21, 0.93]	0.215			
	HL‐PT	4.80 (1.6)	−0.63 (1.2)	−0.43 [−1.00, 0.14]	0.136			
QiEMG_100_ (% MVIC s)	CTR	8.50 (2.5)	0.12 (2.4)	0.05 [−0.15, 0.22]	0.963	1.360	0.252	
	LL‐NE	10.56 (3.1)	−2.23 (3.5)	−0.88 [−1.21, 0.02]	0.531			
	HL‐NE	8.20 (2.7)	0.13 (2.2)	0.05 [−0.57, 0.56]	0.958			
	LL‐PT	8.49 (2.0)	0.23 (2.5)	0.09 [−0.49, 0.64]	0.932			
	HL‐PT	8.24 (2.3)	−0.83 (2.0)	−0.33 [−0.94, 0.19]	0.688			
QiEMG_200_ (% MVIC s)	CTR	15.96 (4.2)	0.09 (3.8)	0.01 [−0.17, 0.19]	0.899	1.217	0.307	
	LL‐NE	18.88 (4.8)	−3.56 (4.8)	−0.64 [−1.24, −0.04]	0.035			
	HL‐NE	15.65 (4.6)	−0.73 (3.3)	−0.20 [−0.76, 0.36]	0.484			
	LL‐PT	16.11 (3.5)	−0.63 (3.9)	−0.15 [−0.7, 0.41]	0.617			
	HL‐PT	15.11 (2.8)	−0.55 (3.6)	−0.20 [−0.76, 0.36]	0.492			

Abbreviations: CI, confidence interval; CTR, control period (note that CTR represents a control period rather than a parallel group); ES, effect size; HL‐NE, heavy‐load power training non‐exercised leg; HL‐PT, heavy‐load power training exercised leg; LL‐NE, light‐load power training non‐exercised leg; LL‐PT, light‐load power training exercised leg; MIF, maximal isometric force; QiEMG, integrated electromyographic signal of the quadriceps; RFD, rate of force development.

### 3.2 1‐RM and *F*–*V* on the Horizontal Leg Press

**FIGURE 2:**
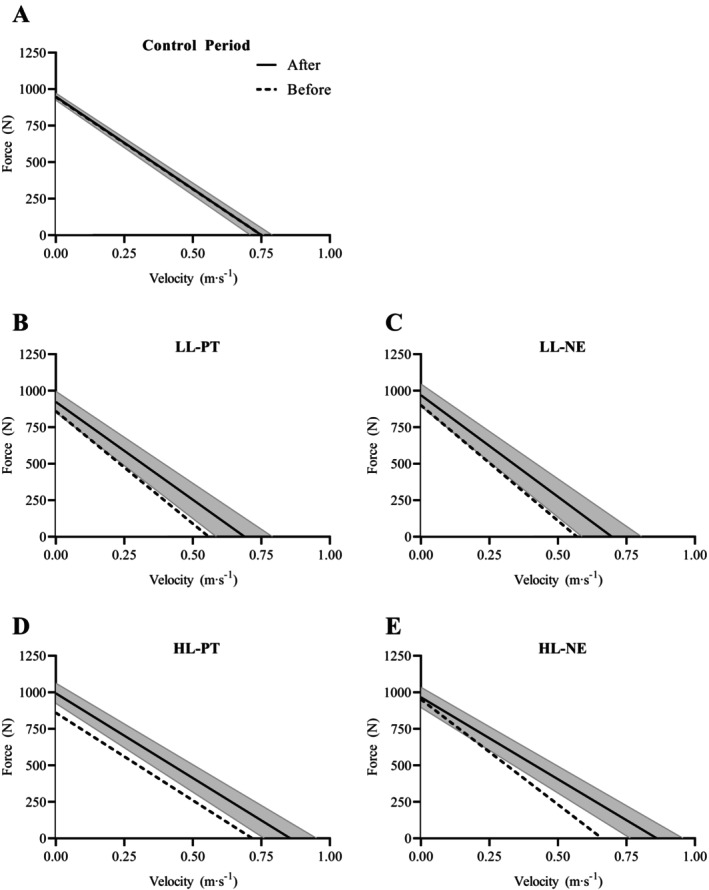
Force‐velocity relationships. Data obtained before (dashed lines) and after (solid lines) the control period (CTR), light‐load power training in the exercised legs (LL‐PT), light‐load power training in the contralateral non‐exercised legs (LL‐NE), heavy‐load power training in the exercised legs (HL‐PT), heavy‐load power training in the contralateral non‐exercised legs (HL‐NE) are shown in panels A, B, C, D and E, respectively. Note that CTR represents a control period rather than a parallel group. The lines represent mean values and the gray areas the corresponding 95% confidence intervals (mean goodness of fit: *R*2 = 0.98 ± 0.02).

The main effects noted in the 1‐RM and parameters derived from the *F*–*V* relationship on the horizontal leg press are shown in Table 3. The effect of treatment on the shape of the *F*–*V* relationship is graphically presented in Figure 2. In summary, both PT interventions (i.e., LL‐PT and HL‐PT) provoked small to moderate increases in 1‐RM (*d* = 0.38–0.65; *p* < 0.001), also observed in the contralateral non‐exercised legs (i.e., LL‐NE and HL‐NE; *d* = 0.35–0.42; *p* < 0.001), but not after CTR (*d* = 0.00; *p* = 0.984). These 1‐RM increments were greater than those after CTR (treatment effect: *F* = 29.805, *p* < 0.001, post hoc: all, *p* < 0.05). Moreover, the 1‐RM increase provoked by HL‐PT was larger than that observed after HL‐NE (post hoc: *p* < 0.05). By contrast, only HL‐PT, triggered a moderate increase of *F*_0_ (*d* = 0.45, *p* < 0.001) that was significantly larger than the change observed after CTR, but similar to the other treatments (treatment effect: *F* = 4.131, *p* = 0.004). Regarding the rapid contractile properties, both PT interventions provoked a moderate increase in *V*_0_ (*d* = 0.45–0.47, *p* ≤ 0.012), also observed in the contralateral non‐exercised legs (*d* = 0.44–0.68, *p* ≤ 0.020), but not after CTR (*d* = −0.02, *p* = 0.675). However, the main effect of treatment for *V*_0_ (*F* = 6.980, *p* < 0.001) and the post hoc comparisons showed that only HL‐PT and HL‐NE were significantly different from CTR (*p* < 0.05). Regarding *P*_max_, a moderate increase was yielded by both PT interventions (*d* = 0.54–0.66, *p* < 0.001), which was also found in the contralateral non‐exercised legs (*d* = 0.49–0.54, *p* < 0.001), whereas no change was observed after CTR (*d* = 0.00, *p* = 0.982). Moreover, these increments in *P*_max_ were significantly different from CTR (main effect of treatment: *F* = 9.554, *p* < 0.001; post hoc: all < 0.001). Finally, no significant differences among treatments were noted in *S*_FV_ (*F* = 0.812, *p* = 0.520).

**TABLE 3:** Baseline values and changes in the horizontal leg press one‐repetition maximum and force‐velocity relationship.

	Treatment	PRE	Change (∆)	Time effect		Treatment effect		
		Mean (SD)	Mean (SD)	ES [95% CI]	*p*	*F*	*p*	Post hoc
1‐RM (kg)	CTR	73.1 (25.1)	−0.2 (6.7)	0.00 [−0.08, 0.08]	0.984	29.805	< 0.001	
	LL‐NE	70.6 (32.2)	11.3 (12.8)	0.42 [0.25, 0.59]	< 0.001			> CTR*
	HL‐NE	71.2 (27.2)	8.0 (7.9)	0.35 [0.20, 0.5]	< 0.001			> CTR*
	LL‐PT	66.3 (19.8)	10.6 (9.2)	0.37 [0.21, 0.53]	< 0.001			> CTR*
	HL‐PT	65.2 (23.8)	16.0 (6.7)	0.64 [0.49, 0.80]	< 0.001			> CTR*; > HL‐NE*
*F*_0_ (*N*)	CTR	945 (298)	3.1 (111)	0.02 [−0.07, 0.10]	0.731	4.131	0.004	
	LL‐NE	900 (365)	83.4 (191)	0.23 [−0.03, 0.49]	0.078			
	HL‐NE	951 (324)	11.4 (83)	0.05 [−0.18, 0.28]	0.692			
	LL‐PT	860 (231)	85.0 (148)	0.21 [−0.03, 0.46]	0.082			
	HL‐PT	859 (286)	138.7 (95)	0.45 [0.21, 0.68]	< 0.001			> CTR*
*V*_0_ (m s^−1^)	CTR	0.75 (0.29)	−0.01 (0.14)	−0.02 [−0.14, 0.09]	0.675	6.980	< 0.001	
	LL‐NE	0.57 (0.11)	0.15 (0.19)	0.46 [0.10, 0.82]	0.020			
	HL‐NE	0.66 (0.24)	0.21 (0.23)	0.70 [0.38, 1.02]	< 0.001			> CTR*
	LL‐PT	0.56 (0.16)	0.15 (0.25)	0.49 [0.14, 0.83]	0.012			
	HL‐PT	0.72 (0.19)	0.14 (0.16)	0.50 [0.18, 0.82]	0.005			> CTR^a^
*P*_max_ (W)	CTR	176 (82)	0.2 (26.1)	0.00 [−0.08, 0.08]	0.982	19.554	< 0.001	
	LL‐NE	130 (61)	43.3 (32.4)	0.54 [0.31, 0.77]	< 0.001			> CTR*
	HL‐NE	159 (86)	38.5 (35.5)	0.48 [0.28, 0.69]	< 0.001			> CTR*
	LL‐PT	123 (52)	40.8 (35.7)	0.53 [0.31, 0.75]	< 0.001			> CTR*
	HL‐PT	155 (70)	53.1 (35.6)	0.66 [0.45, 0.87]	< 0.001			> CTR*
*S*_FV_ [N·(m s^−1^)^−1^]	CTR	−1422 (698)	−9.4 (497)	0.00 [−0.14, 0.13]	0.966	0.812	0.520	
	LL‐NE	−1610 (593)	132.1 (677)	0.12 [−0.34, 0.57]	0.626			
	HL‐NE	−1553 (720)	272.3 (340)	0.34 [−0.07, 0.75]	0.097			
	LL‐PT	−1609 (443)	194.0 (731)	0.20 [−0.22, 0.63]	0.350			
	HL‐PT	−1265 (548)	−62.0 (326)	−0.01 [−0.42, 0.40]	0.968			

Abbreviations: 1‐RM, one‐repetition maximum; CI, confidence interval; CTR, control period (note that CTR represents a control period rather than a parallel group); ES, effect size; *F*_0_, force intercept; HL‐NE, heavy‐load power training non‐exercised leg; HL‐PT, heavy‐load power training exercised leg; LL‐NE, light‐load power training non‐exercised leg; LL‐PT, light‐load power training exercised leg; *P*_max_, maximum muscle power; *S*_FV_, slope of the force‐velocity relationship; *V*_0_, velocity intercept.

* *p* < 0.05.

^a^ *p* < 0.051.

### 3.3 Ultrasound Measurements

**FIGURE 3:**
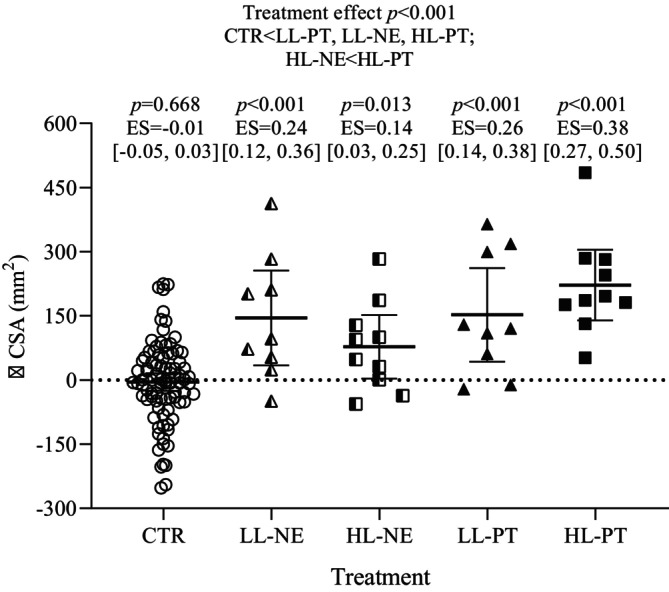
Changes in mid‐thigh muscle cross‐sectional area. Changes (∆) in mid‐thigh muscle cross‐sectional area assessed by ultrasound after the control period (CTR; circles), light‐load power training in the contralateral non‐exercised legs (LL‐NE; mid‐filled triangles), heavy‐load power training in the contralateral non‐exercised legs (HL‐NE; mid‐filled squares), light‐load power training in the exercised legs (LL‐PT, filled triangles) and heavy‐load power training in the exercised legs (HL‐PT; filled squares). Symbols, bars and error bars show individual data, adjusted means and 95% confidence intervals (CI; error bars), respectively, for each treatment. Note that CTR represents a control period rather than a parallel group. The results of statistical tests of between‐group differences in treatment effects are indicated on the top the panel. Within‐group comparisons (time effects) with effect sizes and 95% confidence intervals of changes are shown separately for each treatment.

The changes found in the ultrasound examination are summarized in Figure 3. No significant changes were observed after CTR. In contrast, both PT interventions led to substantial increases in *Q*_CSA_ in the trained limbs compared to CTR (*d* = 0.26–0.39, *p* < 0.001). Additionally, a small but significant increase was observed in the contralateral untrained limbs following LL‐PT (*d* = 0.24, *p* = 0.013) compared to CTR, but not in those following HL‐PT. The overall treatment effect was significant (*F* = 16.94, *p* < 0.001) with post hoc comparisons confirming pairwise differences (all, *p* < 0.05). Notably, the increase in *Q*_CSA_ after HL‐PT was superior to HL‐NE (post hoc: *p* < 0.05).

### 3.4 Functional Performance

**FIGURE 4:**
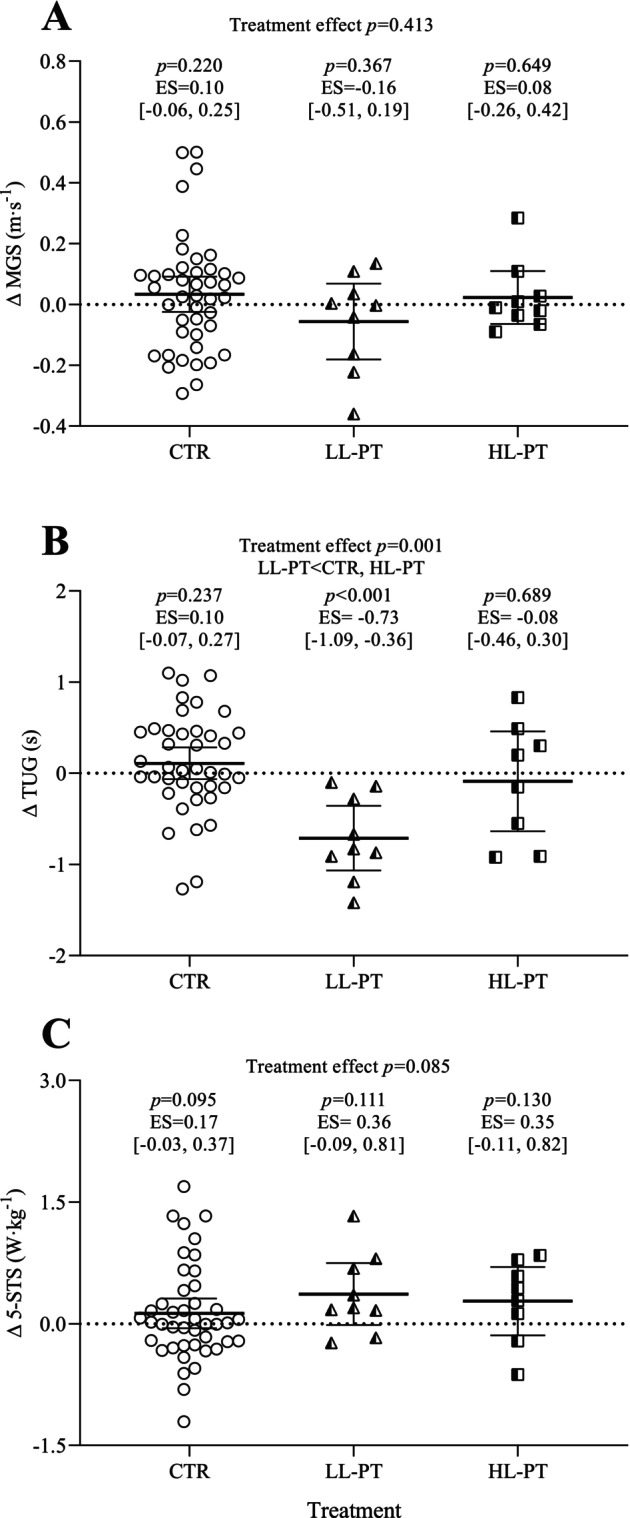
Changes in functional performance. Changes (∆) in maximal gait speed (MGS, panel A), timed‐up and go (TUG, panel B) and estimated power during 5‐repetition sit to stand test (5‐STS, panel C) after the control period (CTR; circles), unilateral light‐load power training (LL‐PT; triangles) and unilateral heavy‐load power training (HL‐PT; squares). Symbols, bars and error bars show individual data, adjusted means and 95% confidence intervals (CI; error bars), respectively, for each treatment. Note that CTR represents a control period rather than a parallel group. The results of statistical tests of between‐group differences in treatment effects are indicated on the top of each panel. Within‐group comparisons (time effects) with effect sizes and 95% confidence intervals of changes are shown separately for each treatment.

The changes noted in functional performance are shown in Figure 4. No significant changes were found in MGS after any of the treatments (*d* = −0.16–0.10, *p* ≥ 0.220; treatment effect: *F* = 0.897, *p* = 0.413). By contrast, large improvements were observed in TUG performance after LL‐PT (*d* = −0.74, *p* < 0.001), compared to HL‐PT and CTR, which remained unchanged (treatment effect: *F* = 8.263, *p* < 0.001; Post hoc: *p* ≤ 0.048). Regarding relative 5‐STS power, no changes were observed after any of the treatments (*d* = 0.17−0.36, *p* ≥ 0.095; treatment effect: *F* = 2.574, *p* = 0.085).

## 4 Discussion

This study found that 12 weeks of unilateral PT for the lower limbs in older adults led to moderate improvements in maximal dynamic strength and *P*_max_ over a non‐exercised contralateral limb, regardless of using LL‐PT or HL‐PT. However, this CE phenomenon was not observed in isometric performance outcomes. Furthermore, only LL‐PT induced a small but significant hypertrophy on the non‐exercised contralateral limb, and enhanced functional performance in a complex task like the TUG test. Since this manuscript is part of a broader project [16], we refer the reader to our previous manuscript in which the effects of intensity on the trained legs are discussed more comprehensively [14].

To the best of our knowledge, this is the first study to analyze the influence of load intensity after unilateral PT on the CE phenomenon in older adults. As a novelty, our study revealed that very different intensities (40% vs. 80% 1RM) applied during PT elicited a similar CE on *P*_max_ in older adults. However, force‐ and velocity‐dependent determinants of muscle power might have contributed differently to the development of *P*_max_. Indeed, HL‐PT but not LL‐PT induced a significant increase in *V*_0_ in the contralateral non‐exercised leg compared to CTR, denoting non‐specific cross adaptations to training velocity (i.e., high‐force, low‐velocity training improved maximal unloaded velocity). This was in line with Hester et al. [12], who observed CE in older adults that improved their torque at high angular velocities (i.e., 300 deg s^−1^) despite training the contralateral leg at low angular velocity (i.e., 45 deg s^−1^) for 4 weeks. In a previous study, we observed greater muscle excitation of the quadriceps muscle (i.e., a proxy of motor unit recruitment and firing rate) during HL‐PT compared to LL‐PT [18]. Thus, considering the intensity of muscle contraction as a strong modulator of the activation of the ipsilateral motor cortex responsible for the control of the untrained limb [25] and its association with *V*_0_ assessed in vivo [26], the heavier loads might be better suited to develop the potential neural adaptations underlying the increase in *V*_0_ of the untrained limb, such as a higher discharge rate [27]. By contrast, within the high‐force, low‐velocity portion of the *F*–*V* relationship, no changes were observed in *F*_0_ of the contralateral non‐exercised legs. A significant CE effect on *F*_0_ could reasonably have been expected following PT, given previous studies demonstrating the CE of MIF after traditional resistance training [28], as well as the strong association between *F*_0_ and MIF [29], both studies conducted in young adults. However, while MIF and *F*_0_ are influenced by common factors (e.g., physiological cross‐sectional area, muscle architecture, fiber type distribution, motor unit recruitment and firing rate) [30], MIF and *F*_0_ should not be considered equivalent due to their methodological differences when collected. In particular, *F*_0_ is derived from multiple maximal dynamic contractions over a wide range of motion compared to MIF which is measured at a fixed muscle length [17]. Moreover, it is widely recognized that the extent of CE is contraction‐type and motor task specific, since the effect is much less when tested in another type of muscle contraction or another exercise, as in our study [7]. Indeed, this was evident in the present study, as there were no changes in isometric performance outcomes like MIF and RFD of the knee extensors following the unilateral PT performed on a horizontal leg press, regardless of the training intensity applied (Table 2). In view of the potential advantages of eccentric training in the effect of CE and more robust transfer to other muscle contraction types compared to traditional resistance training [31], future studies may explore whether including an eccentric overload during PT might warrant additional benefits on isometric performance and *F*_0_ in older adults.

Regarding the CE of maximal dynamic strength, in contrast to our hypothesis, the present study supports that PT induces a similar increase in 1‐RM of the non‐exercised legs, regardless of the PT intensity (mean group change of +11.2% and 16% after HL‐PT and LL‐PT, respectively). Furthermore, the magnitude of the CE‐induced adaptation in 1‐RM found in both groups of our study is comparable to the ~15% increase after resistance training previously reported in a subgroup analysis of older adults included in a meta‐analysis [6]. However, a recent scoping review concluded that the available data consistently demonstrate superior CE in 1‐RM strength when unilateral resistance training is performed at higher versus lower intensities, although most of the studies included were conducted in younger populations [15]. In our study, the greater neuromuscular demands linked to PT compared to traditional RT, especially at moderate to light intensities, might explain the dilution of such between‐load intensities on CE‐induced adaptations in 1‐RM observed after traditional RT. Hence, from a practical point of view, light to moderate intensities can be as effective as heavy intensities for inducing CE adaptations in maximal strength after unilateral RT, as long as maximal intended velocity is applied during the concentric phase.

The ultrasound examinations in our study showed a hypertrophic response in LL‐NE, which was initially unexpected, as most studies have concluded that muscle growth was not a factor underlying the strength gains of CE [32, 33]. However, this study is not the first to show a hypertrophic response in the non‐exercised limb after unilateral resistance training in older adults [34, 35]. It remains unclear whether muscle hypertrophy can result from CE adaptations, and if so, the underlying mechanism of this phenomenon. We hypothesize that possible contributors might include involuntary muscle co‐activation of the non‐exercised limb during training or systemic endocrine responses triggered by unilateral PT. Nevertheless, our findings regarding the hypertrophy of the non‐exercised leg following LL‐PT should be interpreted with caution. None of the participants' limbs were immobilized, and they may have utilized the non‐exercised leg for stabilization while the exercising leg was performing the exercise. Further, specifically designed studies are necessary to elucidate these aspects.

Whether unilateral PT and the corresponding CE response could improve the functional performance of well‐functioning older adults had never been explored. Our results showed that, while no changes were found for relative 5‐STS power and MGS after any of the treatments, only LL‐PT improved TUG performance. Likewise, the high levels of functional performance of our sample (i.e., SPPB score ≈12 points) in addition to the possible ceiling effect of the 5‐STS test [36] and MGS might have clouded the potential benefits of unilateral PT for functional performance, whereas the training effects were evident in the results of the more complex TUG task. The greater contraction velocity inherent to LL‐PT in comparison to HL‐PT may explain the larger effect over TUG performance, since it has been suggested that low‐load, high‐velocity training could be more appropriate to improve functional performance than training at slower contraction velocities [37]. However, this conclusion has been made from studies conducting bilateral PT, so further studies exploring the functional performance after unilateral PT are warranted. Studies involving clinical populations (e.g., stroke, multiple sclerosis, or knee osteoarthritis), in which only the stronger or less involved limb was trained, have shown promising improvements in the overall functional performance in the TUG test or gait speed [35, 38, 39]. However, it is important to note that the CE effects were always lower than those observed in the limb directly trained, so unilateral training should only be considered when training of one limb is not possible due to medical reasons [40]. Therefore, in our opinion, further investigations should explore the effects of the intensity of unilateral PT to minimize the functional performance decline caused by limb immobilization or acute injury in older adults.

Among the limitations of the current manuscript, the degree of co‐activation of the non‐exercised legs was not controlled during the exercise sessions, and thus the CE effects may in part be due to contralateral co‐activation. However, from a clinical point of view, involving the most affected or limited limb as a synergist during the PT of the functional limb might be beneficial, although further research is warranted [40]. Secondly, the CE‐associated adaptations observed in this study cannot be completely ascertained, so further studies should explore the neurophysiological mechanisms. Moreover, since the original sample size calculation was based on a three‐arm design, the exclusion of one arm in the present analysis may have reduced the statistical power. Finally, our study involved well‐functioning older adults without limb immobilization, so the effects of unilateral PT performed to minimize the detrimental consequences of limb immobilization in older adults need to be specifically studied.

In conclusion, unilateral PT of the lower limbs might be suitable to enhance maximal dynamic muscle strength and power of an untrained contralateral limb in older adults independently of the load used. However, the hypertrophic response noted in the untrained contralateral limb and the improvement of functional performance during a complex task in comparison to CTR might point LL‐PT as a more optimal strategy than unilateral HL‐PT in older adults.

## 5 Perspectives

A wide range of intensities during PT can improve muscle and physical function in older adults [13], although these benefits may be underpinned by load‐specific neuromuscular adaptations in the force‐velocity relationship, as shown in our previous study [14]. This secondary analysis builds upon those findings by demonstrating that unilateral PT induces meaningful adaptations in the contralateral untrained lower limb of older adults—specifically in muscle strength and power—regardless of training intensity. These results align with and extend prior research on CE effects in younger populations, suggesting that neuromuscular and morphological benefits can be achieved even in the untrained limb of older adults. Notably, the differential effects of load intensity—where HL‐PT enhanced velocity‐specific adaptations on the untrained limb and LL‐PT uniquely induced hypertrophy of the untrained limb and improved whole‐body physical function—highlight the importance of tailoring PT prescriptions based on individual functional goals. These findings underscore the potential of unilateral PT as a strategic intervention for older adults with unilateral impairments or mobility limitations, offering a non‐invasive means to preserve or enhance muscle function, mass and functional capacity, particularly when using LL‐PT.

## Ethics Statement

Clinical Research Ethics Committee of the Complejo Hospitalario de Toledo (Spain) (ref. 25012017).

## Conflicts of Interest

The authors declare no conflicts of interest.

## Data Availability

The data that support the findings of this study are available from the corresponding author upon reasonable request.
